# Estimation and Prediction of the Polymers’ Physical Characteristics Using the Machine Learning Models

**DOI:** 10.3390/polym16010115

**Published:** 2023-12-29

**Authors:** Ivan Pavlovich Malashin, Vadim Sergeevich Tynchenko, Vladimir Aleksandrovich Nelyub, Aleksei Sergeevich Borodulin, Andrei Pavlovich Gantimurov

**Affiliations:** 1 Artificial Intelligence Technology Scientific and Education Center, Bauman Moscow State Technical University, 105005 Moscow, Russia; vladimir.nelub@emtc.ru (V.A.N.); alexey.borodulin@emtc.ru (A.S.B.); agantimurov@emtc.ru (A.P.G.); 2 Information-Control Systems Department, Institute of Computer Science and Telecommunications, Reshetnev Siberian State University of Science and Technology, 660037 Krasnoyarsk, Russia; 3 Department of Technological Machines and Equipment of Oil and Gas Complex, School of Petroleum and Natural Gas Engineering, Siberian Federal University, 660041 Krasnoyarsk, Russia

**Keywords:** physical characteristics analysis, machine learning, polymers, predictive analytics, random forest, properties

## Abstract

This article investigates the utility of machine learning (ML) methods for predicting and analyzing the diverse physical characteristics of polymers. Leveraging a rich dataset of polymers’ characteristics, the study encompasses an extensive range of polymer properties, spanning compressive and tensile strength to thermal and electrical behaviors. Using various regression methods like Ensemble, Tree-based, Regularization, and Distance-based, the research undergoes thorough evaluation using the most common quality metrics. As a result of a series of experimental studies on the selection of effective model parameters, those that provide a high-quality solution to the stated problem were found. The best results were achieved by Random Forest with the highest $R^{2}$ scores of 0.71, 0.73, and 0.88 for glass transition, thermal decomposition, and melting temperatures, respectively. The outcomes are intricately compared, providing valuable insights into the efficiency of distinct ML approaches in predicting polymer properties. Unknown values for each characteristic were predicted, and a method validation was performed by training on the predicted values, comparing the results with the specified variance values of each characteristic. The research not only advances our comprehension of polymer physics but also contributes to informed model selection and optimization for materials science applications.

## 1. Introduction

The article explores the application of ML techniques in predicting and analyzing the physical characteristics of polymers. Harnessing the power of ML algorithms, the study delves into diverse polymer properties, ranging from compressive and tensile strength to thermal and electrical behavior. The prediction of physical characteristics in polymers is of paramount importance, spanning various industrial and scientific applications. This predictive capability not only enhances our fundamental understanding of polymer behavior [1] but also catalyzes advancements in materials science [2], manufacturing processes [3], and product development [4]. Let us describe the following examples of the polymers’ properties prediction needs:

- **Material Design and Engineering**. Precise predictions of properties such as tensile strength, elasticity, and thermal conductivity empower material scientists in designing polymers with tailored attributes [5]. This facilitates the creation of innovative materials for specific applications, ranging from lightweight composites in aerospace engineering [6] to durable polymers in medical devices [7].
- **Process Optimization.** Understanding and predicting physical characteristics play a crucial role in optimizing manufacturing processes. For instance, predicting melt viscosity in polymer processing aids [8] in controlling the extrusion process, ensuring the production of consistent and high-quality polymer products [9].
- **Quality Control in Polymer Manufacturing**. The ability to predict physical characteristics is instrumental in quality control within polymer manufacturing [10]. Predictive models can assist in identifying deviations in real-time, enabling timely adjustments in the production process to maintain desired material properties.
- **Environmental Impact Assessment**. Predicting properties is essential in determining their biodegradability and recyclability [11]. It contributes to the assessment of a polymer’s environmental impact. This knowledge is particularly relevant in the development of sustainable materials, aligning with the growing emphasis on eco-friendly practices.
- **Pharmaceutical and Medical Applications**. In the field of pharmaceuticals, predicting characteristics can help to determine drug release rates from polymer matrices [12]. It is vital for designing controlled drug delivery systems. Similarly, in medical applications, predicting the mechanical properties of biocompatible polymers is crucial for developing implants and medical devices.

The research employs a variety of regression models, including Lasso Regression [13], Elastic Net [14], Decision Tree Regressor [15], Bagging Regressor [16], AdaBoost Regressor [17], XGBoost Regressor [18], Support Vector Regressor [19], Gradient Boosting Regressor [20], Linear Regression [21], and Random Forest Regressor [22].

Lasso Regression shines in feature selection by inducing sparsity through the regularization of some coefficients to zero [23]. While promoting model simplicity, it does come with the caveat of potentially discarding relevant features and displaying sensitivity to outliers.

Linear Regression, known for its simplicity and interpretability, is suitable for capturing linear relationships [24]. However, its assumption of linearity may limit its performance with intricate, non-linear data. On the other hand, Polynomial Regression, offering flexibility to capture non-linear relationships, is susceptible to overfitting, particularly with higher-degree polynomials.

Support Vector Regression (SVR), effective in high-dimensional spaces and robust to outliers, demands careful selection of kernel and parameters due to its computational intensity [25]. Decision Tree Regression, with its capability to handle non-linearity and interactions, is visually interpretable but prone to overfitting and sensitive to small variations in data.

Random Forest Regression, an ensemble of decision trees, mitigates overfitting but introduces complexity and challenges in interpretation [26].

Gradient Boosting Regression, known for its high predictive accuracy by correcting errors of previous models sequentially, is susceptible to overfitting and requires meticulous hyperparameter tuning [27].

Elastic Net combines the strengths of Lasso and Ridge Regression, offering a balance between feature selection and regularization. However, navigating the optimal mix of L1 and L2 penalties poses a challenge [28].

Decision Tree Regressor excels in capturing non-linear relationships and intricate interactions within the data. Its visual interpretability is a notable asset, but caution is warranted as decision trees are susceptible to overfitting, particularly with complex data [29].

Bagging Regressor, an ensemble technique, mitigates overfitting by aggregating the predictions of multiple decision trees. While enhancing model robustness, it introduces complexity and may be less interpretable [30].

AdaBoost Regressor focuses on sequentially improving model performance by emphasizing misclassified instances. It tends to be less prone to overfitting but is sensitive to noisy data [31].

Gradient Boosting Regressor iteratively builds models, correcting the errors of previous ones [32]. It boasts high predictive accuracy but demands careful parameter tuning to avoid overfitting.

XGBoost Regressor, an extension of Gradient Boosting, excels in predictive accuracy and handles missing data effectively [33]. However, it necessitates careful tuning of hyperparameters and can be computationally intensive.

When generating input for models predicting various physical characteristics of polymers, a diverse set of features such as melting temperature, density and others, and processing conditions are meticulously considered. The inclusion of these multifaceted attributes ensures a comprehensive representation of the intricate relationships governing the polymers’ behavior, enhancing the models’ predictive capabilities.

Each model undergoes rigorous assessment using metrics such as Mean Squared Error [34], R-squared [35], Root Mean Squared Error [36], Normalized Mean Squared Error [37], Mean Absolute Error [38], and Mean Percentage Error [39]. Due to the varying dimensions of the characteristics and the unequal number of non-zero values for each characteristic, it did not make sense to consider Mean Squared Error (MSE) and Mean Absolute Error (MAE). Since Normalized Mean Squared Error (NMSE) is expressed as $1-R^{2}$, only the coefficient of determination ($R^{2}$) and Mean Percentage Error (MPE) were considered as objective metrics. The outcomes are then compared and contrasted, shedding light on the effectiveness of different ML approaches for predicting polymer properties.

The findings not only contribute to advancing the understanding of polymer physics but also offer valuable insights into the selection and optimization of ML models for materials science applications. This research is a significant step towards leveraging ML to enhance our comprehension of complex material behaviors, paving the way for more efficient and accurate predictions in polymer science.

## 2. Materials and Methods

### 2.1. Dataset Preparation

The original dataset contained information on 66,981 different characteristics [40] of polymer materials, representing 18,311 unique polymers with 99 unique physical characteristics, each characterized by varying quantities of known physical attributes [41]. Among these characteristics is crucial information in the form of Simplified Molecular Input Line Entry System (SMILES) strings.

**Figure 1:**
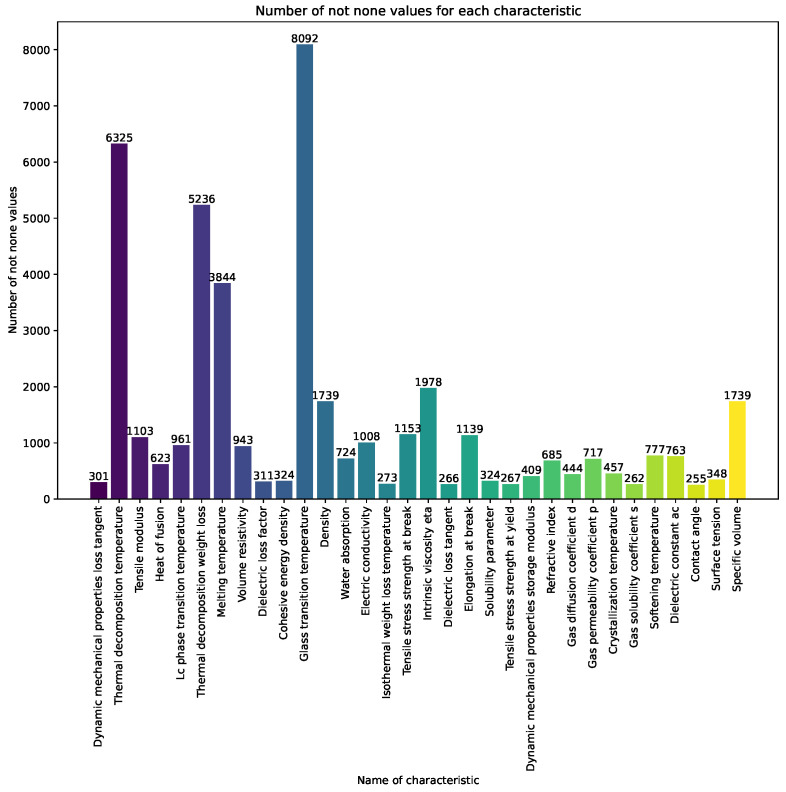
Count of non-null values for each characteristic across the dataset for a count exceeding 250.

**Figure 2:**
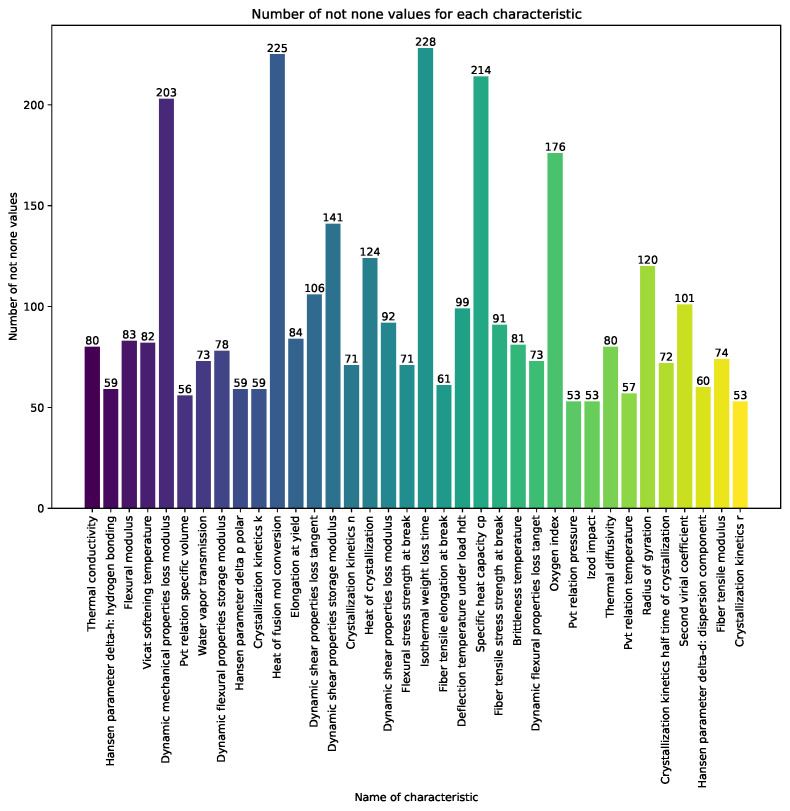
Count of non-null values for each characteristic across the dataset for a count ranging from 50 to 250.

In Figure 1 and Figure 2, the vertical bars represent the count of non-null values for each characteristic across the dataset. The index corresponds to the names of the characteristics, and the vertical axis indicates the count of non-null values. For understanding the completeness of the dataset the numerical annotations on top of each bar provided.

Table A1 and Table A2 provide an overview of key characteristics, including counts, means, standard deviations, minimum and maximum values, medians, and units, offering a comprehensive understanding of the dataset under consideration.

The SMILES strings in the dataset adds a significant dimension to the information available for each polymer material [42]. SMILES provides a standardized and human-readable representation of the chemical structure of molecules. This chemical notation system not only facilitates the accurate identification of distinct polymers but also opens avenues for exploring the relationship between molecular structure and physical characteristics.

**Figure 3:**
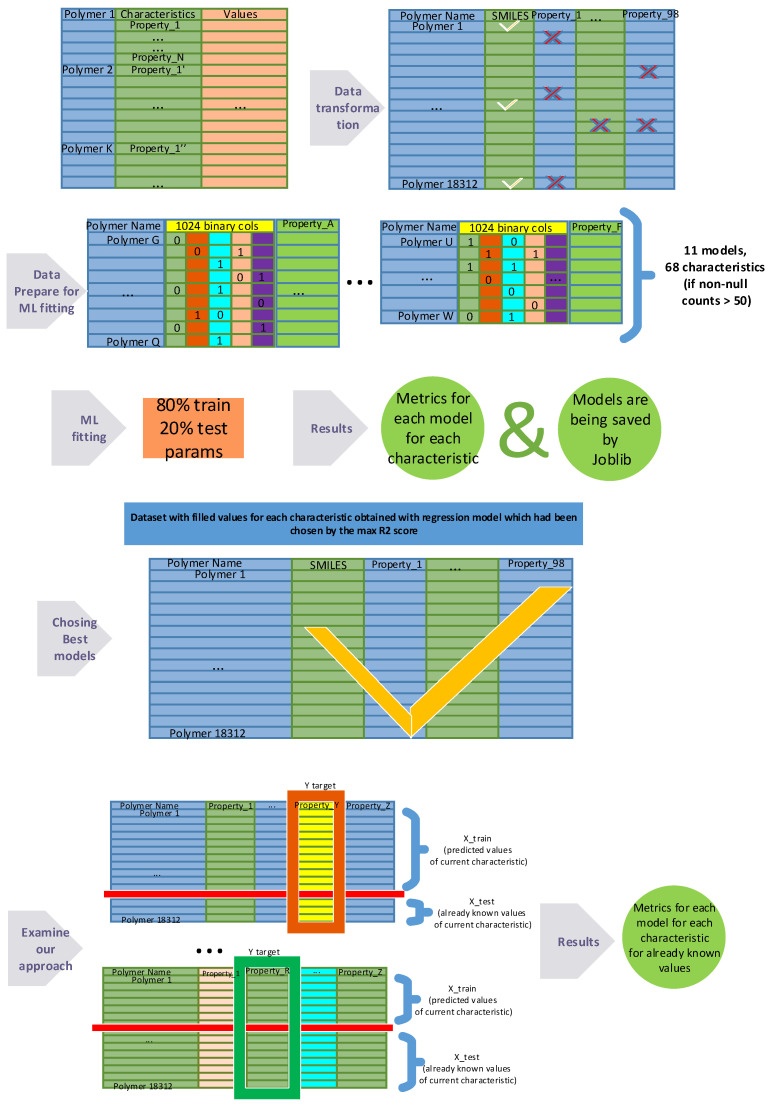
Schematic representation of the dataset transformation process. The original dataset undergoes a series of transformations to create datasets of varying dimensions, each tailored for training on individual physical characteristics. The vectorization involves encoding the SMILES notations into 1024 binary features, facilitating machine learning model training on diverse molecular attributes. Then Prediction Imputation has been used to estimate unknown values for each characteristic. Subsequently, a validation of the method was conducted by training on the predicted values and comparing the outcomes with the specified variance values associated with each characteristic.

The representation of the dataset transformation process is shown in Figure 3.

For each polymer, there was information on the median value of the physical characteristic and the possible variance, although often information about the variance was missing. None of the polymers had complete information on all characteristics.

To initiate the machine learning process, the original dataset underwent a structural transformation. Each row now represents the following structure: the first column contains the material’s name, the second contains the corresponding SMILES string, the third indicates the number of known characteristics for that material, and the fourth lists the names of these characteristics. The subsequent 98 columns contain the median values of all characteristics, and another 98 columns contain the range values for each of these characteristics. This new data structure provides convenience for further analysis and the application of machine learning methods.

The process of vectorizing SMILES into a binary feature vector using RDKit Python library is a crucial step in the analysis of polymer materials [43]. SMILES serves as a string representation of chemical compound structures, and its vectorization is a key stage for applying machine learning methods. To achieve this transformation, a technique is utilized that assigns a unique binary code to each SMILES character. The resulting binary vectors, with a length of 1024, constitute a set of bits reflecting the chemical structure of compounds. This process provides an efficient representation of information about the molecular structure, making it accessible for analysis and processing by machine learning algorithms. Through the vectorization of SMILES, unique numerical representations are created, serving as a valuable tool in addressing tasks related to predicting the physical characteristics of polymers.

### 2.2. Model Training for Predicting the Physical Characteristics of Polymer

In the process of preparing the dataset for predicting the physical characteristics of polymers, multiple transformations were applied to create an optimal data structure. The original dataset, comprising 66,981 unique characteristics of various polymer materials, included information about median values and dispersion. However, this information was often incomplete. To enhance the efficiency of machine learning model training, it was decided to iteratively create new datasets, each consisting of 1024 columns for representing SMILES and an additional column for each physical characteristic containing non-empty values.

Subsequently, each of these created datasets was split into training and testing sets at an 80% to 20% ratio, respectively. In the training phase, diverse machine learning regression models, including but not limited to KNeighborsRegressor, Lasso, Elastic Net, Decision Tree, Bagging, AdaBoost, XGBoost, SVR, Gradient Boosting, Linear Regression, and Random Forest, were utilized to optimize the prediction of physical characteristics in polymer materials. Model performance was evaluated using metrics like MSE (Mean Squared Error), RMSE (Root Mean Squared Error), NMSE (Normalized Mean Squared Error), MAE (Mean Absolute Error), MPE (Mean Percentage Error), $R^{2}$. Additionally, a custom metric was introduced, accounting for the difference between predicted and true values, considering a predefined non-zero dispersion value. The obtained evaluation results enable more effective utilization of trained models for predicting the physical characteristics of polymer materials.

Hyperparameter optimization has been conducted for each model to maximize its predictive capability. Techniques such as grid search, random search to systematically explore the hyperparameter space and identify configurations that yield improved model performance [44].

Subsequently, all the obtained metrics for each feature with post-training on every model were saved in separate files. Following this, a graph analytical processing of these files was conducted to determine the optimal machine learning models for each characteristic.

### 2.3. Using Prediction Method for Imputation of Missing Values of Polymer Physical 98 Characteristics

In contemporary polymer research, extensive datasets of physical characteristics are often analyzed, providing valuable information about material properties. However, the data collection process introduces the challenge of missing values, creating a hurdle in accurately reconstructing the complete dataset. This study introduces a novel approach to address this issue, based on the Prediction Imputation method.

The Prediction Imputation method [45] is a way to fill missing values in data by utilizing machine learning models. In this research, we applied this method to predict missing values for each polymer’s physical characteristic, with the number of missing values varying for each characteristic.

The process involved selecting a suitable machine learning regression model, training it on known data, and then using the trained model to predict values where they were missing. The evaluation of the method included comparing predicted values with real ones, where available.

This innovative approach to handling missing data opens new perspectives for accurate analysis of polymer physical characteristics, improving data recovery and providing more reliable research results.

The analysis of obtained metrics identified optimal regressors for each characteristic, forming a diverse set of best machine learning models. Each applied model was saved using the joblib library for subsequent use.

Subsequently, in accordance with information about the best models, missing values for each characteristic were predicted using the corresponding optimal regressor. These predicted values were merged with the known values, creating a dataset where all characteristics were filled according to the best models used.

Thus, this approach not only efficiently utilizes predictive models for recovering missing data but also allows adapting model selection for each specific characteristic, ensuring more accurate investigation of polymer physical properties.

### 2.4. Examination of Our Approach

To assess the quality of predicted characteristic values, the same series of experiments were conducted to evaluate the consistency between predicted and actual data. For each of the 66 characteristics (for three out of 68 characteristics for which the number of non-zero values was initially greater than 50, the model could not be saved), where the initially known values exceeded 50, an 11-fold experiment was performed.

The specificity of the experiment involved using only predicted values as the training set, while the test set consisted of actually known characteristic values. This approach allowed for evaluating the accuracy of predictive models, considering real data, and provided more reliable indicators than using random or other sample separation methods.

Consistency assessment was conducted using the variance metric. The results of these experiments provide information about the degree of alignment between predicted values and actual data for each regression model, as well as a comprehensive picture across all characteristics.

An important implication of these experiments is the possibility of selecting the most effective models for each specific characteristic, ultimately enhancing the accuracy and reliability of predicting polymer physical property values. The obtained assessments can be utilized to choose optimal regressors for further research in materials science and polymer science.

### 2.5. Categories of Characteristics

The dataset encompasses a diverse array of physical characteristics, each contributing valuable insights into the multifaceted nature of polymer materials. These characteristics are systematically categorized to capture the wide-ranging aspects of a material’s behavior. Compression characteristics and tensile property delve into the material’s response to forces, providing crucial information about its strength and deformability. Creep characteristics illuminate the material’s behavior under sustained loads over time, offering insights into long-term structural integrity. Dilute solution property and rheological property focus on the material’s behavior in solution and its flow properties, respectively, aiding in applications like polymer processing.

The dataset also includes categories such as electric property, shedding light on the material’s conductivity and dielectric properties. Flexural property and shear property offer a nuanced understanding of the material’s response to bending and shearing forces, respectively. Hardness quantifies the material’s resistance to indentation or scratching, while impact strength gauges its ability to absorb sudden impacts. Optical property provides insights into light interaction, and heat characteristics and thermal property delve into the material’s response to temperature changes, including its thermal conductivity and expansion.

**Figure 4:**
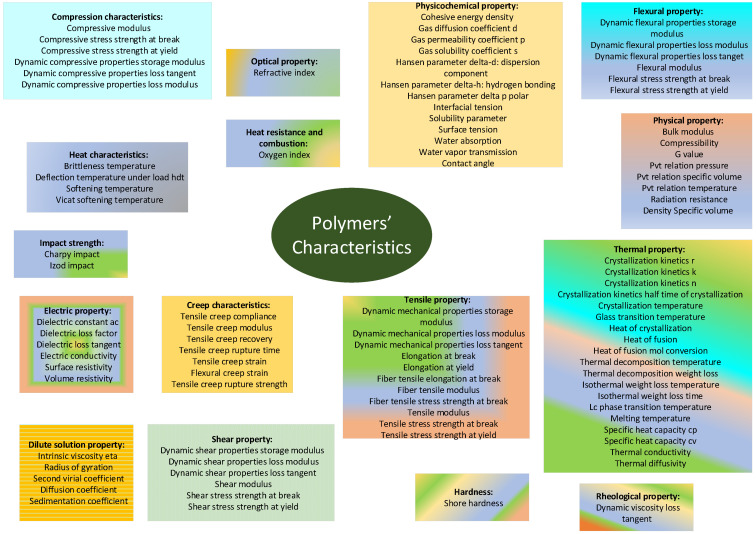
Graph depicting all possible characteristics of physical polymers featured in this study. The characteristics are grouped according to their categories, providing a systematic overview of various aspects of the physical properties of polymer materials.

Heat resistance and combustion characterize the material’s performance under elevated temperatures, contributing to applications where heat stability is crucial. Other physical property and physicochemical property serve as comprehensive categories that encompass a broad spectrum of diverse properties, ensuring a holistic examination. This systematic categorization enhances the dataset’s utility, facilitating targeted exploration and modeling of specific polymer traits for various industrial applications. Figure 4 illustrates the comprehensive spectrum of physical polymer characteristics explored in this study.

The characteristics are systematically arranged based on their respective categories, offering a structured representation of diverse aspects of polymer material properties. The description of each physical characteristic presented in the dataset is provided in Appendix B.

## 3. Results

Experimental conditions involved transforming SMILES representations into binary features and training models individually on each characteristic using non-empty values. The experiment utilized an Intel(R) Core(TM) i7-7700 CPU @ 3.60 GHz for computational tasks [46].

**Figure 5:**
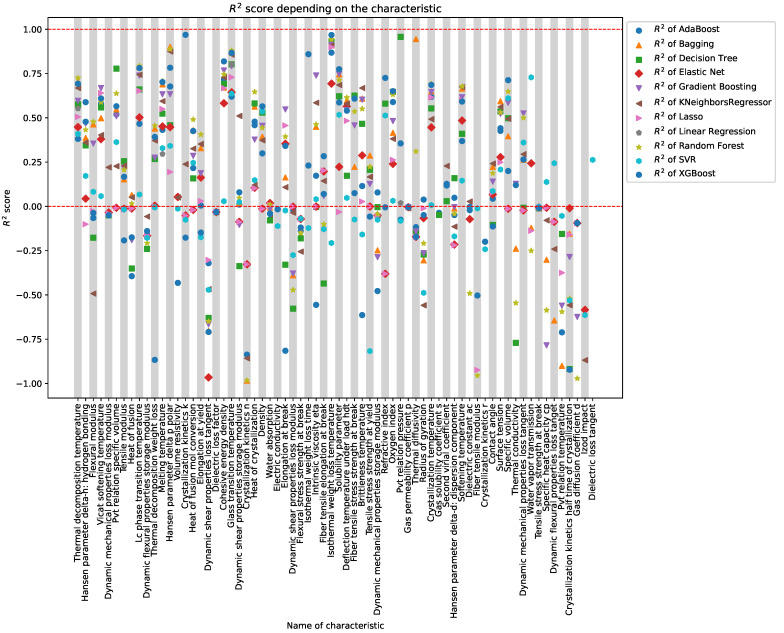
Array of $R^{2}$ Scores, each point is a testament to the mastery of machine learning models in deciphering the intricacies of physical traits.

In Figure 5, $R^{2}$ scores are illustrated for 68 characteristics, each of which has more than 50 non-zero values in the original dataset. Thirty-one (31) characteristics exhibit $R^{2}$ values within the range of 0.5 to 1.

The alignment of optimal metric values across all regression models for each characteristic highlights a consistent pattern. This alignment emphasizes the robust performance of machine learning models in predicting physical characteristics of polymers, particularly for the identified subset of characteristics. The coherence in results across various models underscores the reliability and effectiveness of the chosen models in capturing the underlying patterns in the dataset.

Optimal regression models and metrics for physical characteristics are shown in Table 1. The table presents the most effective regression models and associated metrics for predicting various physical characteristics of polymers. Each row corresponds to a specific characteristic, showcasing the selected regression model, the maximum $R^{2}$ score achieved, and the corresponding Normalized Mean Squared Error (NMSE). The models were carefully evaluated, and the results offer insights into the predictive performance for different characteristics in the polymer dataset.

**Figure 6:**
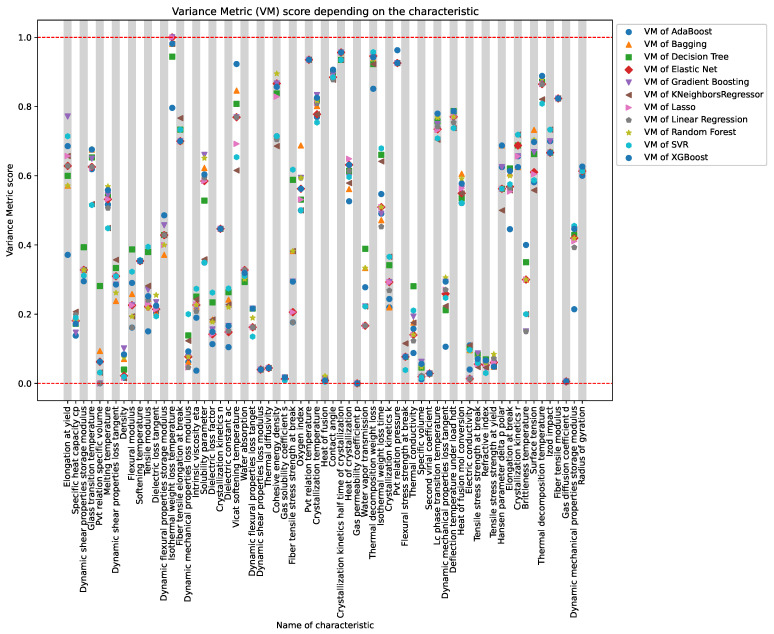
Array of VM Scores, each point is a testament to the mastery of machine learning models in deciphering the intricacies of physical traits.

Figure 6 depicts a graph of variance metric values for all initially known characteristics of polymers. Different characteristics are marked on the *x*-axis, while the *y*-axis displays the values of the variance metric, measuring the degree of correspondence between predicted and actual values. Each stripe on the graph corresponds to one of the 66 characteristics, where the initial number of known values exceeds 50.

The presented graph allowing for a visual assessment of the predictive models’ effectiveness for each specific characteristic. Stripes rising above indicate high accuracy in predictions, while those descending below may suggest some disparities between predicted and real values.

Table 2 presents optimal variance metrics obtained by training on predicted values and subsequently predicting known values for validation, demonstrating the model’s performance on the dataset.

## 4. Discussion

In recent years, there has been substantial interest in accelerating materials design and discovery, spurred by initiatives like the Materials Genome Initiative and Integrated Computational Materials Engineering [47]. This perspective aims to outline general problems, information science methods, and outstanding challenges in the field of materials informatics [48]. For example, ref. [49] introduces Polymer Genome [50], a web-based machine-learning capability for near-instantaneous predictions of polymer properties. Study [51] explores computational alternatives, Group Interaction Modeling (GIM), and Machine Learning (ML), for predicting thermal and mechanical properties of polymers. The paper [52] addresses challenges in utilizing machine learning for polymer discovery, focusing on accurately representing complex, multi-scale structures. Ref. [53] introduces a promising CGCNN framework that directly learns material properties from crystal structures, offering a universal and interpretable representation. The paper [54] addresses the ongoing debate in molecular property prediction by comparing two prominent classes of models—neural networks applied to computed molecular fingerprints or expert-crafted descriptors and graph convolutional neural networks. The authors of [55] introduce a data-driven framework for predicting work functions of complex compounds, showcasing the effectiveness of a random forest model in achieving high accuracy. The paper [56] contributes significantly to the chemistry machine learning field by showcasing the potential of machine learning methods in predicting bulk properties of molecules, specifically crystalline density.

In this study, we observed distinct patterns in the performance of regression models across various characteristics, particularly focusing on the glass transition temperature, thermal decomposition temperature, and melting temperature, which exhibited substantial counts of non-null values (3844, 6325, and 8092, respectively).

This study presents an evaluation of various regression methods. The study does not merely apply ML algorithms but goes further, conducting experimental studies to select the best model for each physical characteristic. This meticulous approach showcases a commitment to refining models for improved predictive accuracy, providing valuable insights into the strengths and weaknesses of different ML approaches.

For characteristics with a higher number of non-null values, we noticed a proportional increase in the $R^{2}$ score as the data size expanded. Specifically, the $R^{2}$ scores for glass transition temperature, thermal decomposition temperature, and melting temperature were 0.71, 0.73, and 0.88, respectively. Random Forest emerged as the optimal regression model for these characteristics, showcasing its ability to handle larger datasets and capture complex relationships. These scores serve as a baseline for future work and highlight the inherent strengths and limitations of each regressor in its default configuration.

On the other hand, for characteristics with a data size ranging from 176 to 2000, XGBoost and Gradient Boosting demonstrated superior performance, outshining other regression models. These findings suggest that these boosting algorithms excel in capturing intricate patterns within datasets of a moderate size.

Interestingly, for characteristics with lower data sizes, ranging from 59 to 141, a mix of regression models, including Decision Tree, Bagging, KNeighborsRegressor, AdaBoost, and SVR, displayed competitive performance. The diversity in optimal models for these characteristics implies that the choice of the most suitable regression algorithm may depend on the specific characteristics of the dataset, and a one-size-fits-all approach might not be appropriate.

Several factors could contribute to these observations. Firstly, the complexity of the relationship between molecular features (captured by SMILES strings) and physical characteristics may vary across different characteristics, influencing the model’s performance. Additionally, the nature of the dataset, including the distribution of non-null values and the diversity of polymer structures, might impact the effectiveness of certain regression models. Further investigations into the specific molecular features contributing to the predictive power of each model and a deeper understanding of the underlying chemical processes could provide valuable insights into the observed patterns.

The results of the analysis of the variance metric presented in Table 2 yielded insightful observations regarding the performance of predictive models based solely on predicted values derived from optimal regressors. Surprisingly, a significant alignment was observed between the majority of characteristics and the models, resulting in notably high variance metric scores. This consistency suggests a robust predictive capability of the chosen regressors across various physical properties of polymers.

Several factors contribute to the success of the models, while also shedding light on potential pitfalls. Characteristics exhibiting exceptionally high values, such as volume resistivity, might present challenges in prediction due to their intrinsic variability or non-linear dependencies on other factors. Additionally, features with substantial data dispersion or limited data points may introduce uncertainties, influencing the precision of predictions.

The remarkable congruence between characteristics and models implies a certain universality in the efficacy of the selected regressors. The results underscore the adaptability of these models across diverse physical attributes of polymers, enhancing their utility in materials science research.

However, it is essential to acknowledge that the success of predictive modeling is contingent on the nature of the characteristic being predicted. While the variance metric serves as a comprehensive metric, its applicability can be context-dependent. High metric scores indicate successful prediction, but the interpretation should consider the specific challenges associated with each characteristic.

Depending on the size of the dataset, different models are considered the best fit for various physical properties of polymers. This variation in model suitability can be attributed to the complex and heterogeneous nature of polymer systems [57]. Large datasets may facilitate the application of more complex models, such as ensemble methods or deep learning, to capture intricate relationships [58], while smaller datasets may benefit from simpler models to avoid overfitting [59].

The reasons for the different natures of models include the distribution of data [60], the presence of non-linearities and interactions in physical properties [61], and the dimensionality of the feature space. In high-dimensional feature spaces, models like Lasso Regression or Elastic Net may be preferred for feature selection and regularization, while simpler models like linear regression may suffice for fewer features.

Similar phenomena are observed in other systems such as colloids [62], proteins [63], and nucleic acids [64]. The optimal choice of models for predicting physical descriptors varies based on the nature of the system and the characteristics of the data.

- **Colloids:** different models may be suitable for predicting properties such as particle size, shape, and stability, considering the diverse interactions and conditions influencing colloidal systems [65].
- **Proteins:** the structure and function of proteins may require distinct modeling approaches. For example, machine learning models like Random Forests may be effective for predicting protein-ligand binding affinities [66], while simpler models may suffice for secondary structure prediction [67].
- **Nucleic Acids:** the unique properties of nucleic acids, such as DNA or RNA, may demand different models for predicting structural features [68], interaction energies, or other physical descriptors based on the specific characteristics of the dataset.

The discussions highlight both the achievements and challenges encountered in using prediction imputation for estimating missing values in polymer physical characteristics. The positive alignment of characteristics and models indicates the promising potential of this approach, opening avenues for further refinement and application in the field of materials science and polymer research.

## 5. Conclusions

In conclusion, this study aimed to predict missing values for various physical characteristics of polymers using machine learning techniques. The predictive models, including Random Forest, Gradient Boosting, and XGBoost, demonstrated strong performance, with the Random Forest model achieving the highest $R^{2}$ scores of 0.71, 0.73, and 0.88 for glass transition temperature, thermal decomposition temperature, and melting temperature, respectively. The validation process involved predicting unknown values, showcasing the reliability of the models.

The best-performing model, Random Forest, displayed promising results in handling the complexity of polymer characteristics. Future research could explore refining the models further, considering additional feature engineering, and expanding the dataset to enhance predictive accuracy. Additionally, investigating the transferability of the models to different polymer datasets could contribute to the broader applicability of the developed predictive framework. Some directions for further research are:

1. **Feature Engineering and Selection**: explore advanced feature engineering techniques and refine feature selection methods to identify the most influential characteristics. Investigate the impact of incorporating domain-specific knowledge to enhance the models’ ability to capture subtle nuances in polymer behavior.
2. **Model Optimization**: this includes experimenting with different ensemble methods, regularization techniques, and model architectures to achieve a more robust and accurate predictive framework.
3. **Dataset Expansion**: consider augmenting the dataset by incorporating data from diverse polymer sources. A larger and more diverse dataset could provide a comprehensive understanding of polymer characteristics, enabling models to generalize better across different types of polymers.
4. **Cross-Dataset Validation**: evaluate the transferability of the developed models by validating them on external polymer datasets. Assessing the models’ performance on different datasets will provide insights into their robustness and applicability across various polymer compositions and properties.
5. **Incorporating Temporal Aspects**: if applicable, consider incorporating temporal aspects into the models to capture any time-dependent trends or changes in polymer characteristics. This could involve analyzing how polymers evolve over time under different conditions.
6. **Interpretability and Explainability**: enhance the interpretability of the models to provide clearer insights into the features driving predictions. This could involve employing techniques such as SHAP (SHapley Additive exPlanations) values to explain the contribution of each feature to the model’s output.
7. **Uncertainty Quantification**: integrate methods for uncertainty quantification to provide more reliable predictions and confidence intervals. This is particularly important in applications where understanding the uncertainty associated with predictions is crucial for decision-making.
8. **Collaboration with Domain Experts**: foster collaboration between data scientists and domain experts in polymer science to gain deeper insights into the underlying physics and chemistry. Leveraging domain knowledge can lead to the development of more informed models and a better understanding of the relationships between polymer characteristics.

By addressing these avenues, future research endeavors can contribute to the continuous improvement and applicability of machine learning models in predicting and understanding the complex characteristics of polymers.

## Figures and Tables

**Table 1:** Optimal regression models and $R^{2}$ best scores for physical characteristics.

Characteristic	Data Size ^1^	Best Regressor	Max $R^{2}$	MPE
Glass transition temperature	8092	Random Forest	0.88	1.23
Thermal decomposition temperature	6325	Random Forest	0.73	2.25
Melting temperature	3844	Random Forest	0.71	1.05
Intrinsic viscosity ETA	1978	Gradient Boosting	0.74	
Specific volume	1739	XGBoost	0.71	2.75
Density	1739	XGBoost	0.56	0.5
Elongation at break	1139	Gradient Boosting	0.55	
LC phase transition temperature	961	Random Forest	0.79	3.02
Softening temperature	777	Random Forest	0.68	20.73
Refractive index	685	XGBoost	0.73	0.91
Crystallization temperature	457	Random Forest	0.69	6.3
Surface tension	348	Bagging	0.59	0.06
Solubility parameter	324	XGBoost	0.77	0.04
Cohesive energy density	324	XGBoost	0.82	0.96
Dynamic mechanical properties loss tangent	301	Gradient Boosting	0.52	
Isothermal weight loss temperature	273	XGBoost	0.97	0.13
Isothermal weight loss time	228	XGBoost	0.86	
Oxygen index	176	XGBoost	0.65	12.24
Dynamic shear properties storage modulus	141	KNeighborsRegressor	0.51	
Heat of crystallization	124	Random Forest	0.65	
Deflection temperature under load HDT	99	Random Forest	0.61	4.0
Fiber tensile stress strength at break	91	Decision Tree	0.63	1.1
Vicat softening temperature	82	Gradient Boosting	0.67	0.45
Brittleness temperature	81	KNeighborsRegressor	0.67	1.2
Thermal diffusivity	80	Bagging	0.94	4.13
Water vapor transmission	73	SVR	0.73	
Hansen parameter delta p polar	59	Bagging	0.9	0.45
Hansen parameter delta-h: hydrogen bonding	59	AdaBoost	0.59	2.56
Crystallization kinetics k	59	XGBoost	0.97	
PVT relation specific volume	56	Decision Tree	0.78	0.01
PVT relation pressure	53	Decision Tree	0.96	

^1^ The reported data size corresponds to the count of non-none values for each median parameter for each characteristic.

**Table 2:** Optimal regression models and best variance metrics (VM) scores for physical characteristics.

Characteristic	Data Size ^1^	Best Regressor	Max VM
Isothermal weight loss temperature	219	Elastic Net	1.0
PVT relation pressure	26	AdaBoost	0.96
Thermal decomposition weight loss	3567	SVR	0.96
Crystallization kinetics half time of crystallization	26	AdaBoost	0.96
PVT relation temperature	26	AdaBoost	0.94
Vicat softening temperature	56	Random Forest	0.92
Contact angle	116	Random Forest	0.91
Cohesive energy density	219	Random Forest	0.9
Thermal decomposition temperature	2968	XGBoost	0.89
Crystallization temperature	331	Gradient Boosting	0.83
Fiber tensile modulus	40	AdaBoost	0.82
Deflection temperature under load HDT	38	Bagging	0.79
LC phase transition temperature	430	XGBoost	0.78
Elongation at yield	49	Gradient Boosting	0.77
Fiber tensile elongation at break	31	KNeighborsRegressor	0.77
Izod impact	23	KNeighborsRegressor	0.73
Surface tension	176	Bagging	0.73
Crystallization kinetics r	21	KNeighborsRegressor	0.72
Oxygen index	144	Bagging	0.69
Hansen parameter delta p polar	43	Bagging	0.69
Isothermal weight loss time	175	SVR	0.68
Glass transition temperature	6278	Random Forest	0.68
Solubility parameter	218	Gradient Boosting	0.66
Heat of crystallization	67	Lasso	0.65
Radius of gyration	45	Bagging	0.63
Elongation at break	854	Decision Tree	0.62
Fiber tensile stress strength at break	57	SVR	0.62
Heat of fusion mol conversion	154	Bagging	0.61
Melting temperature	2182	Random Forest	0.57

^1^ The reported data size corresponds to the count of non-null values for each variance parameter for each characteristic.

## Data Availability

All code, datasets, and images referenced in this article are publicly available in the following GitHub repository: https://github.com/catauggie/polymersML (accessed on 24 November 2023). Researchers are encouraged to refer to this repository for access to the complete set of resources used in the study.

## Appendix A. Data Description

**Table A1:** Summary of physical characteristics (more than 300 values).

Characteristic	Count	Mean	Std	Min	Max	50%	Unit
Dynamic mechanical properties loss tangent	301	0.56	1.12	0.0	11.6	0.14	
Thermal decomposition temperature	6325	401.0	112.87	18.0	1000.0	403.0	C
Tensile modulus	1103	3.69	13.03	0.0	202.0	2.1	GPa
Heat of fusion	623	0.01	0.01	0.0	0.12	0.01	kcal/g
LC phase transition temperature	961	191.76	95.37	−90.0	528.0	175.0	C
Thermal decomposition weight loss	5236	10.25	13.09	0.0	100.0	5.0	%
Melting temperature	3844	194.93	108.24	−54.0	580.0	186.35	C
Volume resistivity	943	$1.15\times 10^{16}$	$1.21\times 10^{17}$	0.0	$3.1\times 10^{18}$	$45\times 10^{8}$	ohm·cm
Dielectric loss factor	311	775.51	13608.97	0.0	240000.0	0.1	
Cohesive energy density	324	112.21	60.3	0.0	626.0	96.0	cal/cm^3^
Glass transition temperature	8092	145.18	110.69	−123.0	495.0	138.0	C
Density	1739	1.24	0.2	0.23	3.03	1.23	g/cm^3^
Water absorption	724	10.95	48.98	0.0	1065.0	2.5	wt%
Electric conductivity	1008	$1.96\times 10^{6}$	$61.73\times 10^{6}$	0.0	$19.6\times 10^{9}$	0.0	1/(ohm·cm)
Elongation at break	1139	51.98	157.26	0.26	3000.0	10.1	%
Tensile stress strength at break	1153	0.19	2.14	0.0	64.02	0.08	GPa
Intrinsic viscosity ETA	1978	1.43	12.4	0.0	495.0	0.52	dl/g
Solubility parameter	324	21.08	5.0	0.0	51.2	20.0	(J/cm^3^)${}^{1/2}$
Dynamic mechanical properties storage modulus	409	2.28	4.58	0.0	64.6	1.3	GPa
Refractive index	685	1.65	0.86	0.49	23.0	1.6	
Gas diffusion coefficient d	444	0.0	0.0	0.0	0.0	0.0	cm^2^/s
Gas permeability coefficient p	717	0.0	0.0	0.0	0.0	0.0	cm^3^(STP)cm/(cm^2^·s·Pa)
Crystallization temperature	457	138.4	105.61	−120.0	496.0	124.0	C
Softening temperature	777	176.31	103.88	−185.0	800.0	173.0	C
Dielectric constant AC	763	22.51	403.72	0.12	11,002.15	3.26	
Surface tension	348	30.95	13.08	5.75	72.5	31.13	mN/m
Specific volume	1739	0.83	0.15	0.33	4.3	0.81	cm^3^/g
Dielectric loss tangent	266.0	0.74	4.8	−0.03	55.0	0.02	
Isothermal weight loss temperature	273.0	389.35	165.13	100.0	900.0	350.0	C
Tensile stress strength at yield	267.0	0.07	0.05	0.0	0.4	0.06	GPa
Contact angle	255.0	73.96	19.85	15.0	158.9	76.0	degree
Gas solubility coefficient s	262.0	0.01	0.06	0.0	0.69	0.0	cm^3^ (STP)/cm^3^·Pa)

**Table A2:** Summary of Physical Characteristics (More than 50 up to 250 Values).

Characteristic	Count	Mean	Std	Min	Max	50%	Unit
Thermal conductivity	80	0.81	2.95	0.01	23.0	0.22	W/(m·K)
Hansen parameter delta−h: hydrogen bonding	59	8.03	3.5	0.0	16.0	7.4	(J/cm${}^{3}{)}^{1/2}$
Flexural modulus	83	8.27	21.18	0.04	108.0	2.61	GPa
Vicat softening temperature	82	137.08	59.47	29.7	380.0	133.0	C
Dynamic mechanical properties loss modulus	203	2.47	22.2	0.0	260.0	0.1	GPa
PVT relation specific volume	56	0.85	0.17	0.4	1.17	0.85	cmcm^3^/g
Water vapor transmission	73	0.82	2.38	0.0	15.0	0.01	g·mil/(cm^2^·24 h)
Dynamic flexural properties storage modulus	78	1.71	4.36	0.0	37.0	0.79	GPa
Hansen parameter delta p polar	59	7.11	4.84	1.1	19.5	6.1	(J/cm${}^{3}{)}^{1/2}$
Crystallization kinetics k	59	0.66	2.25	0.0	15.07	0.01	
Heat of fusion mol conversion	225	3.99	3.33	0.0	21.0	3.32	kcal/mol
Elongation at yield	84	22.45	50.18	0.08	334.0	8.3	%
Dynamic shear properties loss tangent	106	1.88	14.6	0.0	150.0	0.07	
Dynamic shear properties storage modulus	141	0.43	0.67	0.0	3.65	0.03	GPa
Crystallization kinetics n	71	2.59	0.72	0.59	4.15	2.6	
Heat of crystallization	124.0	10.39	9.95	0.29	49.3	8.3	cal/g
Dynamic shear properties loss modulus	92	0.05	0.11	0.0	0.7	0.0	GPa
Flexural stress strength at break	71	0.15	0.29	0.0	1.84	0.09	GPa
Isothermal weight loss time	228	86.9	333.57	0.18	2500.0	13.8	h
Fiber tensile elongation at break	61	39.65	48.71	2.25	242.34	21.0	%
Deflection temperature under load HDT	99	189.38	87.48	45.0	417.0	197.0	C
Specific heat capacity CP	214	0.38	0.25	0.0	2.52	0.35	cal/(g·C)
Fiber tensile stress strength at break	91	50.8	329.54	0.17	3090.0	3.6	g/denier
Brittleness temperature	81	−22.15	35.05	−80.0	90.0	−26.0	C
Dynamic flexural properties loss tanget	73	0.59	0.71	0.0	3.02	0.17	
Oxygen index	176	35.85	14.05	4.5	95.0	34.0	%
PVT relation pressure	53	74.91	135.54	0.0	598.8	35.0	MPa
Izod impact	53	161.43	350.27	0.02	1990.0	40.0	kJ/m
Thermal diffusivity	80	0.0	0.0	0.0	0.0	0.0	m^2^/s
PVT relation temperature	57	216.95	523.81	4.0	3822.0	87.5	C
Radius of gyration	120	33.15	36.39	0.5	264.35	21.72	nm
Crystallization kinetics half time of crystallization	72	2389.53	6064.79	11.1	35,400.0	289.5	s
Second virial coefficient	101	0.15	1.07	−0.0	8.95	0.0	cm^3^·mol/g^2^
Hansen parameter delta−d: dispersion component	60	16.54	4.09	0.0	21.5	17.53	(J/cm${}^{3}{)}^{1/2}$
Fiber tensile modulus	74	90.59	156.26	3.86	847.0	43.5	g/denier
Crystallization kinetics r	53	1986.31	10,850.65	0.02	79,175.0	97.0	nm/s

## Appendix B. Physical Characteristics

### Appendix B.1. Physical Properties

1. **Bulk Modulus:** measures a material’s resistance to volume change under pressure. It is crucial for understanding how a material responds to changes in pressure [69].
2. **Compressibility:** describes the degree to which a material can be compressed. It is the reciprocal of bulk modulus and helps assess a material’s response to external pressure [70].
3. **G Value:** represents the ratio of the strain energy stored in a material to the kinetic energy. It provides insights into a material’s elastic behavior under deformation [71].
4. **PVT Relation Pressure:** describes the relationship between pressure and specific volume in a material. It is essential for understanding the material’s response to changes in pressure and volume [72].
5. **PVT Relation Specific Volume:** defines the correlation between specific volume and pressure in a material. It is crucial for analyzing the material’s behavior under varying pressure conditions [73].
6. **PVT Relation Temperature:** illustrates the relationship between temperature and specific volume in a material. It is essential for studying how temperature influences the material’s volume properties [74].
7. **Radiation Resistance:** measures a material’s ability to withstand the effects of ionizing radiation. This property is vital for materials used in radiation-exposed environments [75].
8. **Density:** represents the mass of a material per unit volume. Density is a fundamental property that influences various material characteristics [76].
9. **Specific Volume:** describes the volume occupied by a unit mass of a material. It is the reciprocal of density and provides insights into material compactness [77].

### Appendix B.2. Compression Characteristics

1. **Compressive Modulus:** measures the material’s resistance to compression. Essential in the construction of structural elements made of polymers [78].
2. **Compressive Stress Strength at Break:** determines the maximum pressure a polymer can withstand before breaking. Important for assessing the resilience of polymer structures to mechanical forces [79].
3. **Compressive Stress Strength at Yield:** measures the strength of a polymer under pressure before plastic deformation begins. Important for the preliminary evaluation of the material’s structural reliability [80].
4. **Dynamic Compressive Properties Storage Modulus:** characterizes the material’s ability to store energy under dynamic loading. Important for materials subjected to cyclic loads, such as in damping materials [81].
5. **Dynamic Compressive Properties Loss Tangent:** reflects the fraction of energy loss due to dynamic deformation. Important in the development of materials with effective damping properties [82].
6. **Dynamic Compressive Properties Loss Modulus:** determines the energy loss during dynamic deformation. Important for materials designed for sound absorption or vibration reduction [83].

### Appendix B.3. Creep Characteristics

1. **Tensile Creep Compliance:** determines the polymer’s ability to undergo deformation under constant tensile load. This is crucial for assessing the long-term stability of polymer materials under constant force or load [84].
2. **Tensile Creep Modulus:** measures the elasticity of the polymer when deformed under constant force. This parameter is useful in designing materials for applications where resistance to constant mechanical loads is important [85].
3. **Tensile Creep Recovery:** evaluates the polymer’s ability to return to its original shape after deformation under tensile loading. This is important, for example, for materials used in springs or elastic elements [86].
4. **Tensile Creep Rupture Time:** specifies the period during which the polymer undergoes deformation before rupture under tensile loading. This is an important characteristic for assessing the material’s resistance to long-term mechanical loads [87].
5. **Tensile Creep Strain:** measures the level of deformation a polymer can undergo under constant tensile force. This is important for understanding the material’s behavior under constant load and can be used in the design of structural elements [88].
6. **Flexural Creep Strain:** evaluates the deformation of the polymer under constant load during bending. This characteristic is important, for example, when using polymer materials in structures subjected to constant bending forces [89].
7. **Tensile Creep Rupture Strength:** determines the maximum load a polymer can withstand before rupture under constant tensile force. This is a crucial parameter for assessing the durability and resilience of polymer materials under constant mechanical loads [90].

### Appendix B.4. Dilute Solution Property

1. **Intrinsic Viscosity (η):** measures the polymer’s resistance to flow in a dilute solution, providing insights into its molecular size and structure. Intrinsic viscosity is crucial for understanding the polymer’s solubility and processing behavior [91].
2. **Radius of Gyration:** defines the average distance of polymer segments from the center of mass, indicating the spatial extent of the polymer chain in solution. This property is significant in studying polymer conformations [92].
3. **Second Virial Coefficient:** describes the non-ideality of polymer solutions, providing information about the intermolecular interactions and solute-solvent interactions. This coefficient influences the solution behavior and phase separation [93].
4. **Diffusion Coefficient:** represents the rate at which polymer molecules spread through the solution, influencing mass transport and the polymer’s ability to interact with its surroundings [94].
5. **Sedimentation Coefficient:** measures the rate at which polymer particles settle under the influence of gravity in a centrifugal field, providing information about particle size and shape in solution [95].

### Appendix B.5. Electric Property

1. **Dielectric Constant (AC):** reflects the material’s ability to store electrical energy in an alternating current (ac) field. The dielectric constant influences the capacitance of electronic components [96].
2. **Dielectric Loss Factor:** measures the efficiency with which a dielectric material converts electrical energy into heat. This property is crucial in applications where minimal energy loss is desired [97].
3. **Dielectric Loss Tangent:** describes the ratio of the dielectric loss factor to the dielectric constant, providing insights into the material’s efficiency in handling electrical energy [98].
4. **Electric Conductivity:** represents the ability of a material to conduct electric current. This property is essential in various electronic and electrical applications [99].
5. **Surface Resistivity:** defines the electrical resistance across the surface of a material, influencing its performance in applications where surface conductivity is critical [100].
6. **Volume Resistivity:** measures the electrical resistance through the volume of a material, providing information about its overall resistance to electric current flow [101].

### Appendix B.6. Flexural Property

1. **Dynamic Flexural Properties Storage Modulus:** characterizes the material’s ability to store energy under dynamic flexural (bending) loading conditions. Important for materials subjected to cyclic loads [102].
2. **Dynamic Flexural Properties Loss Modulus:** determines the energy dissipation capacity of the material during dynamic flexural deformation. Relevant for applications requiring effective damping [103].
3. **Dynamic Flexural Properties Loss Tangent:** reflects the ratio of the loss modulus to the storage modulus in dynamic flexural deformation, providing insights into the material’s damping behavior [104].
4. **Flexural Modulus:** measures the material’s stiffness and resistance to bending deformation. Crucial in designing structural components where flexural strength is essential [105].
5. **Flexural Stress Strength at Break:** indicates the maximum stress a material can withstand before fracturing under bending stress. Important for evaluating the material’s structural integrity [106].
6. **Flexural Stress Strength at Yield:** measures the material’s stress resistance under bending before exhibiting plastic deformation. Important for assessing structural reliability under flexural loads [107].

### Appendix B.7. Hardness

1. **Shore Hardness:** measures the resistance of the material to indentation or penetration. Shore hardness is a valuable indicator of a material’s overall hardness and durability [108].

### Appendix B.8. Heat Characteristics

1. **Brittleness Temperature:** indicates the temperature at which a material transitions from a flexible to a brittle state, providing insight into its low-temperature performance [109].
2. **Deflection Temperature under Load (HDT):** represents the temperature at which a standard test bar experiences a specified deformation under a specific load. HDT is crucial for understanding a material’s ability to withstand elevated temperatures while supporting a load [110].
3. **Softening Temperature:** defines the temperature range at which a material starts to soften, losing its rigidity. Softening temperature is essential for assessing a material’s behavior under heat [111].
4. **Vicat Softening Temperature:** determines the temperature at which a needle penetrates a material under a specified load. Vicat softening temperature provides insights into the heat resistance and stability of a material [112].

### Appendix B.9. Heat Resistance and Combustion

1. **Oxygen Index:** measures the minimum concentration of oxygen in a mixture with an inert gas that supports the combustion of a material. This parameter is crucial for evaluating a material’s fire resistance and combustion characteristics [113].

### Appendix B.10. Impact Strength

1. **Charpy Impact:** assesses a material’s resistance to sudden impact by measuring the amount of energy absorbed during fracture. Charpy impact testing is widely used to evaluate the toughness of materials [114].
2. **Izod Impact:** similar to Charpy impact testing, Izod impact testing measures a material’s resistance to impact. It assesses the energy required to break a notched specimen under a sudden impact [115].

### Appendix B.11. Optical Property

1. **Refractive Index:** determines the degree to which light is refracted or bent as it passes through a material. Refractive index is essential for understanding optical transparency and performance in various applications [116].

### Appendix B.12. Physicochemical Property

1. **Cohesive Energy Density:** represents the energy required to separate unit volumes of material. It is a measure of the cohesive forces within a substance [117].
2. **Gas Diffusion Coefficient (*D*):** describes the rate at which gas molecules diffuse through a substance. It is crucial for understanding gas transport properties [118].
3. **Gas Permeability Coefficient (*P*):** measures a material’s ability to allow gas permeation. It is essential for applications where gas barrier properties are significant [119].
4. **Gas Solubility Coefficient (*S*):** represents the capacity of a material to dissolve gases. This property is vital for understanding gas absorption in polymers [120].
5. **Hansen Parameter δ−d: Dispersion Component:** describes the dispersion forces within a material. It is part of the Hansen solubility parameters, which characterize solute-solvent interactions [121].
6. **Hansen Parameter δ−h: Hydrogen Bonding:** represents the hydrogen bonding contribution to the Hansen solubility parameters. It provides insights into materials’ compatibility with various solvents [122].
7. **Hansen Parameter δ−p: Polar:** describes the polar forces within a material. It is another component of the Hansen solubility parameters [123].
8. **Interfacial Tension:** measures the energy required to increase the surface area between two phases. It is crucial for understanding interactions at material interfaces [124].
9. **Solubility Parameter:** represents the overall solubility characteristics of a substance. It is a combination of the Hansen parameters and is used to predict material compatibility [125].
10. **Surface Tension:** describes the force acting on the surface of a liquid that tends to minimize the area. Surface tension is vital for understanding wetting and adhesion [126].
11. **Water Absorption:** measures the ability of a material to absorb water. It is essential for assessing the material’s response to humid environments [127].
12. **Water Vapor Transmission:** describes the rate at which water vapor permeates through a material. It is crucial for applications requiring water vapor barrier properties [128].
13. **Contact Angle:** represents the angle formed between a liquid droplet and a solid surface. It provides insights into the wettability of a material [129].

### Appendix B.13. Rheological Property

1. **Dynamic Viscosity Loss Tangent:** describes the ratio of the loss modulus to the storage modulus in the context of dynamic viscosity. It provides insights into the energy dissipation behavior of the material under dynamic conditions [130].

### Appendix B.14. Shear Property

1. **Dynamic Shear Properties Storage Modulus:** represents the ability of a material to store elastic energy under shear stress in dynamic conditions [131].
2. **Dynamic Shear Properties Loss Modulus:** describes the portion of energy that a material loses as heat under shear stress in dynamic conditions [132].
3. **Dynamic Shear Properties Loss Tangent:** represents the ratio of the loss modulus to the storage modulus in the context of dynamic shear properties. It provides insights into the material’s response to shear forces [133].
4. **Shear Modulus:** measures a material’s resistance to deformation under shear stress. It is crucial for understanding a material’s shear behavior [134].
5. **Shear Stress Strength at Break:** represents the maximum shear stress a material can withstand before experiencing failure. It is an essential parameter for evaluating the material’s shear strength [135].
6. **Shear Stress Strength at Yield:** measures the shear stress a material can withstand before undergoing plastic deformation. This parameter is crucial for assessing the material’s yield strength under shear forces [136].

### Appendix B.15. Tensile Property

1. **Dynamic Mechanical Properties Storage Modulus:** represents the material’s ability to store elastic energy under dynamic tensile conditions [137].
2. **Dynamic Mechanical Properties Loss Modulus:** describes the portion of energy that a material loses as heat under dynamic tensile conditions [138].
3. **Dynamic Mechanical Properties Loss Tangent:** represents the ratio of the loss modulus to the storage modulus in the context of dynamic tensile properties. It provides insights into the material’s response to dynamic tensile forces [139].
4. **Elongation at Break:** measures the extent to which a material can stretch before experiencing rupture. It is a crucial parameter for evaluating the material’s ductility [140].
5. **Elongation at Yield:** measures the material’s deformation before it starts yielding under tensile stress. This parameter provides insights into the material’s yield behavior under tension [141].
6. **Fiber Tensile Elongation at Break:** describes the elongation capability of fiber materials before experiencing rupture under tensile stress [142].
7. **Fiber Tensile Modulus:** represents the stiffness of a fiber material under tensile stress. It is a critical parameter for assessing the material’s tensile rigidity [143].
8. **Fiber Tensile Stress Strength at Break:** represents the maximum tensile stress a fiber material can withstand before undergoing rupture [144].
9. **Tensile Modulus:** measures the material’s resistance to deformation under tensile stress. It is crucial for understanding the material’s tensile behavior [145].
10. **Tensile Stress Strength at Break:** represents the maximum tensile stress a material can withstand before experiencing failure [146].
11. **Tensile Stress Strength at Yield:** measures the tensile stress a material can withstand before undergoing plastic deformation. This parameter is crucial for assessing the material’s yield strength under tensile forces [147].

### Appendix B.16. Thermal Property

1. **Crystallization Kinetics r:** characterizes the crystallization kinetics of a material, representing the rate of crystallization [148].
2. **Crystallization Kinetics k:** represents a parameter in the crystallization kinetics equation, providing insights into the crystallization process [149].
3. **Crystallization Kinetics n:** another parameter in the crystallization kinetics equation, influencing the rate of crystallization [150].
4. **Crystallization Kinetics Half Time of Crystallization:** describes the time required for half of the crystallization process to occur [151].
5. **Crystallization Temperature:** represents the temperature at which a material undergoes crystallization [152].
6. **Glass Transition Temperature:** indicates the temperature at which an amorphous material transitions from a rigid to a rubbery state [153].
7. **Heat of Crystallization:** represents the amount of heat released or absorbed during the crystallization process [154].
8. **Heat of Fusion:** describes the heat energy required to change a substance from a solid to a liquid state at a constant temperature [155].
9. **Heat of Fusion Mol Conversion:** provides insights into the heat energy required for the conversion of a mole of substance from solid to liquid state [156].
10. **Thermal Decomposition Temperature:** represents the temperature at which a material starts to decompose thermally [157].
11. **Thermal Decomposition Weight Loss:** describes the weight loss associated with the thermal decomposition of a material [158].
12. **Isothermal Weight Loss Temperature:** represents the temperature maintained during a process where a material experiences weight loss [159].
13. **Isothermal Weight Loss Time:** describes the duration of time during which a material undergoes weight loss under isothermal conditions [160].
14. **LC Phase Transition Temperature:** represents the temperature at which a phase transition occurs in the liquid crystalline state [161].
15. **Melting Temperature:** indicates the temperature at which a material transitions from a solid to a liquid state [162].
16. **Specific Heat Capacity $C_{p}$:** describes the amount of heat energy required to raise the temperature of a unit mass of a material by one degree Celsius at constant pressure [163].
17. **Specific Heat Capacity $C_{v}$:** similar to $C_{p}$ but at constant volume, representing the heat energy required to raise the temperature at constant volume [164].
18. **Thermal Conductivity:** describes the ability of a material to conduct heat [165].
19. **Thermal Diffusivity:** represents the ability of a material to conduct heat relative to its ability to store heat. It is the ratio of thermal conductivity to volumetric heat capacity [166].
